# Protocol for a type 3 hybrid implementation cluster randomized clinical trial to evaluate the effect of patient and clinician nudges to advance the use of genomic medicine across a diverse health system

**DOI:** 10.1186/s13012-024-01385-5

**Published:** 2024-08-19

**Authors:** Anna C. Raper, Benita L. Weathers, Theodore G. Drivas, Colin A. Ellis, Colleen Morse Kripke, Randall A. Oyer, Anjali T. Owens, Anurag Verma, Paul E. Wileyto, Colin C. Wollack, Wenting Zhou, Marylyn D. Ritchie, Robert A. Schnoll, Katherine L. Nathanson

**Affiliations:** 1 Division of Translational Medicine and Human Genetics, Department of Medicine, Perelman School of Medicine, University of Pennsylvania, 3400 Civic Center Blvd, Philadelphia, PA 19104 USA; 2 Department of Neurology, Perelman School of Medicine, University of Pennsylvania, Philadelphia, PA USA; 3 Division of Cardiology, Department of Medicine, Perelman School of Medicine, University of Pennsylvania, Philadelphia, PA USA; 4 Department of Genetics, Perelman School of Medicine, University of Pennsylvania, Philadelphia, PA USA; 5 Division of Biostatistics, Department of Biostatistics, Epidemiology and Informatics, Perelman School of Medicine, University of Pennsylvania, Philadelphia, PA USA; 6 Abramson Cancer Center, Perelman School of Medicine, University of Pennsylvania, Philadelphia, PA USA; 7 Center for Interdisciplinary Research on Nicotine Addiction, University of Pennsylvania, Philadelphia, PA USA; 8 Information Services Applications, Penn Medicine, University of Pennsylvania, Philadelphia, PA USA

**Keywords:** Genetic testing, Behavioral economics, Nudges, Implementation science, Electronic health record, Pragmatic, Type 3 hybrid cluster randomization trial

## Abstract

**Background:**

Germline genetic testing is recommended for an increasing number of conditions with underlying genetic etiologies, the results of which impact medical management. However, genetic testing is underutilized in clinics due to system, clinician, and patient level barriers. Behavioral economics provides a framework to create implementation strategies, such as nudges, to address these multi-level barriers and increase the uptake of genetic testing for conditions where the results impact medical management.

**Methods:**

Patients meeting eligibility for germline genetic testing for a group of conditions will be identified using electronic phenotyping algorithms. A pragmatic, type 3 hybrid cluster randomization study will test nudges to patients and/or clinicians, or neither. Clinicians who receive nudges will be prompted to either refer their patient to genetics or order genetic testing themselves. We will use rapid cycle approaches informed by clinician and patient experiences, health equity, and behavioral economics to optimize these nudges before trial initiation. The primary implementation outcome is uptake of germline genetic testing for the pre-selected health conditions. Patient data collected through the electronic health record (e.g. demographics, geocoded address) will be examined as moderators of the effect of nudges.

**Discussion:**

This study will be one of the first randomized trials to examine the effects of patient- and clinician-directed nudges informed by behavioral economics on uptake of genetic testing. The pragmatic design will facilitate a large and diverse patient sample, allow for the assessment of genetic testing uptake, and provide comparison of the effect of different nudge combinations. This trial also involves optimization of patient identification, test selection, ordering, and result reporting in an electronic health record-based infrastructure to further address clinician-level barriers to utilizing genomic medicine. The findings may help determine the impact of low-cost, sustainable implementation strategies that can be integrated into health care systems to improve the use of genomic medicine.

**Trial registration:**

ClinicalTrials.gov. NCT06377033. Registered on March 31, 2024. https://clinicaltrials.gov/study/NCT06377033?term=NCT06377033&rank=1

**Supplementary Information:**

The online version contains supplementary material available at 10.1186/s13012-024-01385-5.

Contributions to the literature

- This paper describes the development of implementation strategies and electronic phenotyping to support an implementation clinical trial focused on genetic testing.
- This study will evaluate the use of implementation strategies informed by behavioral economics to promote uptake of genetic testing in a group of conditions for which the results may change medical management.
- This study may identify novel supports to reduce disparities and increase uptake of genetic testing in patients with pre-selected conditions.

## Background

The number of conditions for which genetic testing is indicated, as we understand more about how results influence medical management for patients, is increasing exponentially [1–3]. However, only a minority of patients with these conditions receive genetic testing and benefit from the use of genomic medicine [4]. Oncology specialties have been early adopters of germline genetic testing, and currently the identification of pathogenic variants in high and moderate penetrance cancer predisposition genes leads to critical changes in clinical management, including treatment selection [5, 6]. In other specialties, including cardiology, neurology, and endocrinology, the use of genetic testing to guide medical management is quickly being adopted with broad use across multiple diseases [2, 7–13]. Genetic testing now is recognized as an essential part of clinical care, with payers exhibiting a strong preference in approving coverage of genetic tests that change medical care [14] and patients recognizing the potential benefits of precision medicine [15, 16]. Despite this recognition, the literature demonstrates a considerable practice gap in implementation of genetic testing for many conditions including neuroendocrine tumors [17], myopathies, amyotrophic lateral sclerosis [ALS], cardiomyopathies [18, 19], long QT syndrome [20], hereditary amyloidosis, and other cardiovascular conditions [21–24]. Beyond the underutilization of genomic medicine generally, substantial disparities in genetic testing use exist (as has been demonstrated for minority patients with breast and ovarian cancer [25] and neurologic conditions [26]). Thus, novel and sustainable approaches to enable genetic testing for patients with conditions in which the results change medical care must be developed and implemented.

Barriers to the use of genomic medicine across medical specialties exist at the system, clinician, and patient levels. With over 50,000 genetic tests now available, and the list of indications for genetic testing greatly increasing, the demand cannot be met by the existing clinical genetics workforce [27, 28]. Infrastructure development to support delivery of genomics care is needed to increase the number of providers who can help to facilitate such care [29]. There are also issues in the current system of genomic medicine of data flow, including practical challenges with integrating genomic data into the electronic health record (EHR), such as the size and complexity of genetic test results, inadequate use of standards for genetic data, and a lack of widespread clinical decision support (CDS) [30, 31].

For non-geneticist providers (such as physician or advanced practice provider [APP]), multiple barriers hinder the implementation of genomics in routine care including: lack of knowledge about the conditions for which genetic testing is indicated, accompanying medical management implications, how to proceed if there is recognition that testing is needed, and concerns about cost and patient reaction [29, 32–35]. Although there is agreement that the first step towards increasing efficiency in genetic testing is a more automated way to identify high risk patients, few algorithms exist to do so, and they are not focused on conditions for which genetic testing will influence medical management [36]. Strategies to improve patient identification, optimization of test selection, and ordering and result reporting in an EHR-based infrastructure can address the clinician level barriers to genomic medicine and broaden its scope [37–40]. A clinician’s recommendation is an influential determinant of whether patients receive genetic testing [41, 42]. Nevertheless, factors including patient awareness of genetic testing beliefs, attitudes, and individual emotional responses, interpersonal relationships, and access to genetic clinics also serve as barriers to genetic testing [12, 42]. Patients also express concerns about potential costs and adverse consequence of testing such as stigma, discrimination, emotional distress, or harms to family members [11, 42–44]; many of these barriers are exacerbated in racial and ethnic minority patients [45–47]. Therefore, processes directed at patient action are also needed to maximize the utilization of genomic medicine. Implementation science addresses these critical practice gaps using rigorous scientific methods to understand why evidence-based treatments are not utilized and what strategies effectively promote utilization [48]. It is well-recognized that there is critical need to merge implementation science with genomic medicine, including by NHGRI and a National Academies of Sciences, Engineering, and Medicine workgroup [49, 50].

A growing body of research from behavioral economics suggests that limits in thinking capacity, available information, and time can impair rational decision making [51]. As decision makers, people are influenced by a myriad of psychological, social, cognitive, and emotional factors, and use simplifying cognitive heuristics biases [52, 53]. Status quo bias, or the tendency to stick with a current approach even if new or better approaches are available, especially plays a role in use of evidence-based interventions [54–57]. Studies across multiple healthcare areas have shown promising results when applying this insight to modify physician behavior [58–62]. Implementation strategies informed by behavioral economics can increase the use of evidence-based treatment, as shown for recommending tobacco use treatment [63–66], promoting the use of serious illness conversations [67], and improving use of higher value prescribing [68]. More recently, groups have started to evaluate behavioral economic constructs as barriers to physician use of genetic testing [69].

One of the most powerful ways to affect behavior is through nudges in the EHR, which involve making subtle changes to the way that choices are presented [70, 71]. Nudges improved the use of evidence-based treatments in 73% of studies in a review [72]. When the evidence-based intervention requires both clinician and patient actions, as for genetic testing, a multi-level intervention that nudges both stakeholder groups may be required. Although not extensively evaluated, studies have shown that nudges directed towards patients that included default for engagement with an evidence-based intervention (e.g., diabetes self-monitoring and medication adherence following myocardial infarction) significantly increased engagement with the targeted health behavior [66, 73–75]. One type of patient-directed nudge is priming, where information is conveyed to patients directly to activate willingness and interest, and counteract common barriers regarding an evidence-based treatment or health behavior [76]. Priming previously has been shown to increase the use of serious illness conversations and decrease overuse of medications [77–79].

**Fig. 1:**
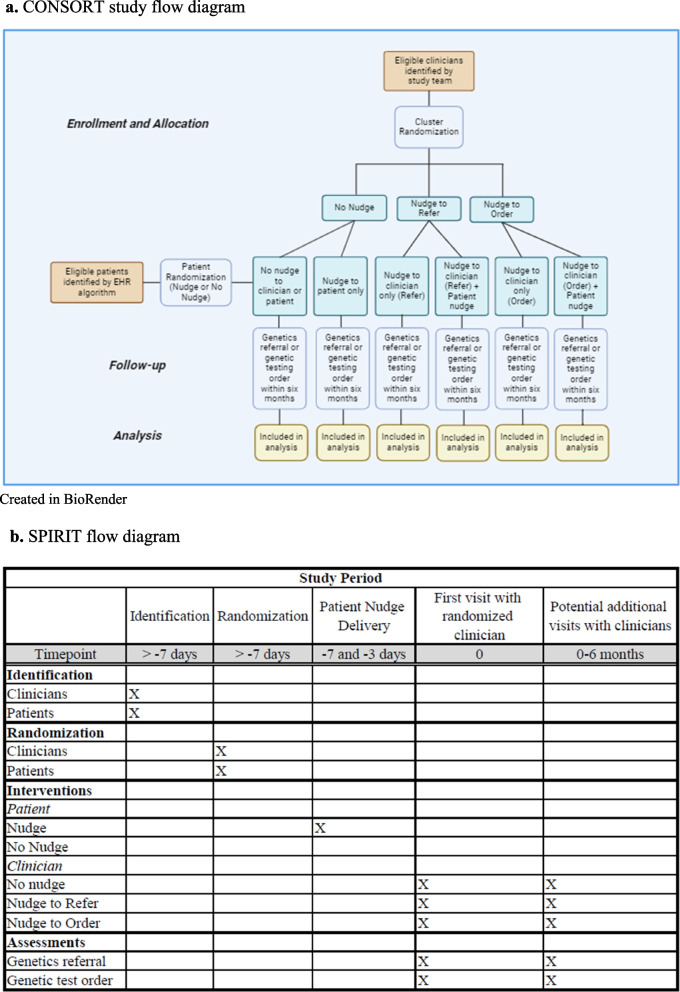
**a** CONSORT study flow diagram Created in BioRender. **b** SPIRIT flow diagram

This study aims to refine clinician- and patient-directed implementation strategies informed by behavioral economics to evaluate the effect on uptake of genomic medicine. Electronic phenotyping algorithms [80] will first be defined to identify patients for whom genetic testing is recommended. Nudges will be designed to address clinician and patient heuristics that can influence decision-making within complex medical care. We will conduct a hybrid type 3 cluster-randomized controlled trial (RCT) to evaluate these optimized patient- and/or clinician-directed strategies for increasing the use of genetic testing in conditions for which the results inform medical management. Given ongoing awareness of health disparities in genomic medicine [81, 82], and the shift in focus of health equity in implementation science [83–85], impact of these strategies in racial minorities will be evaluated. Methods and resources developed in this trial will then be disseminated through Penn websites (Fig. 1) and public websites.

## Methods

### Study design

**Fig. 2:**
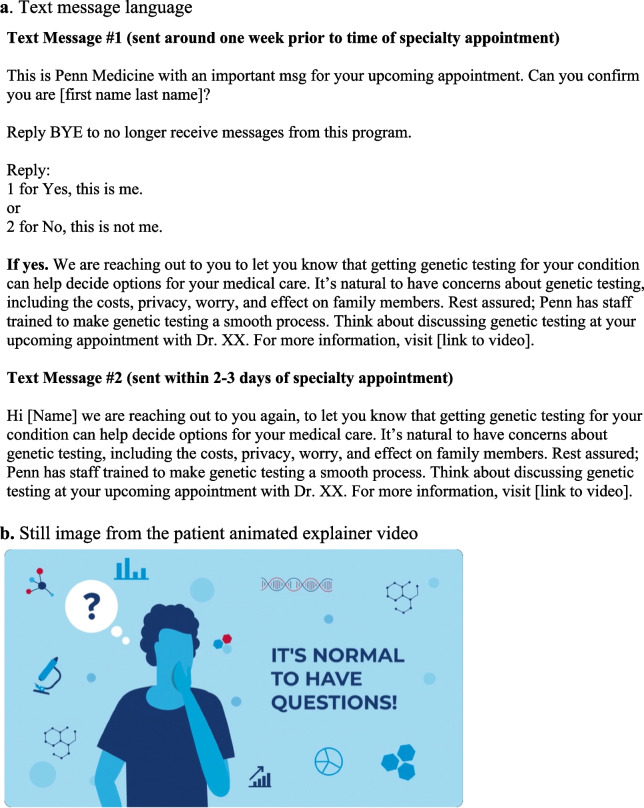
Patient Nudges. **a** Text message language. **b** Still image from the patient animated explainer video

The study will test optimized implementation strategies informed by behavioral economics in a six-arm factorial hybrid type 3 cluster implementation RCT testing the effectiveness of nudges to clinicians (to either refer to a genetics clinic or order genetic testing), nudges to patients (to discuss genetic testing with their clinician), nudges to both, or neither (Fig. 2). The two versions of the clinician nudge will help to test the effects of a local adaptation to this implementation strategy.

Primary and secondary implementation outcomes, and contextual factors that shape implementation effectiveness (e.g., clinician location, patient demographics) will be captured. Prior to the nudges being sent out, electronic phenotyping algorithms will be used to identify patients for whom genetic testing is recommended based on published guidelines and expert opinion [2, 3, 7, 10]. Conditions were selected for which 1) genetic testing is considered standard of care due to implications for medical management; 2) the genetic testing approach is standardized; and 3) effective electronic phenotyping with a positive predictive value (PPV) [39, 86] of over 85% can be performed (Supplemental Table 1). With a lens of health equity, preliminary data also were collected for these medical conditions highlighting existing disparities in genetic testing rates by race.

**Fig. 3:**
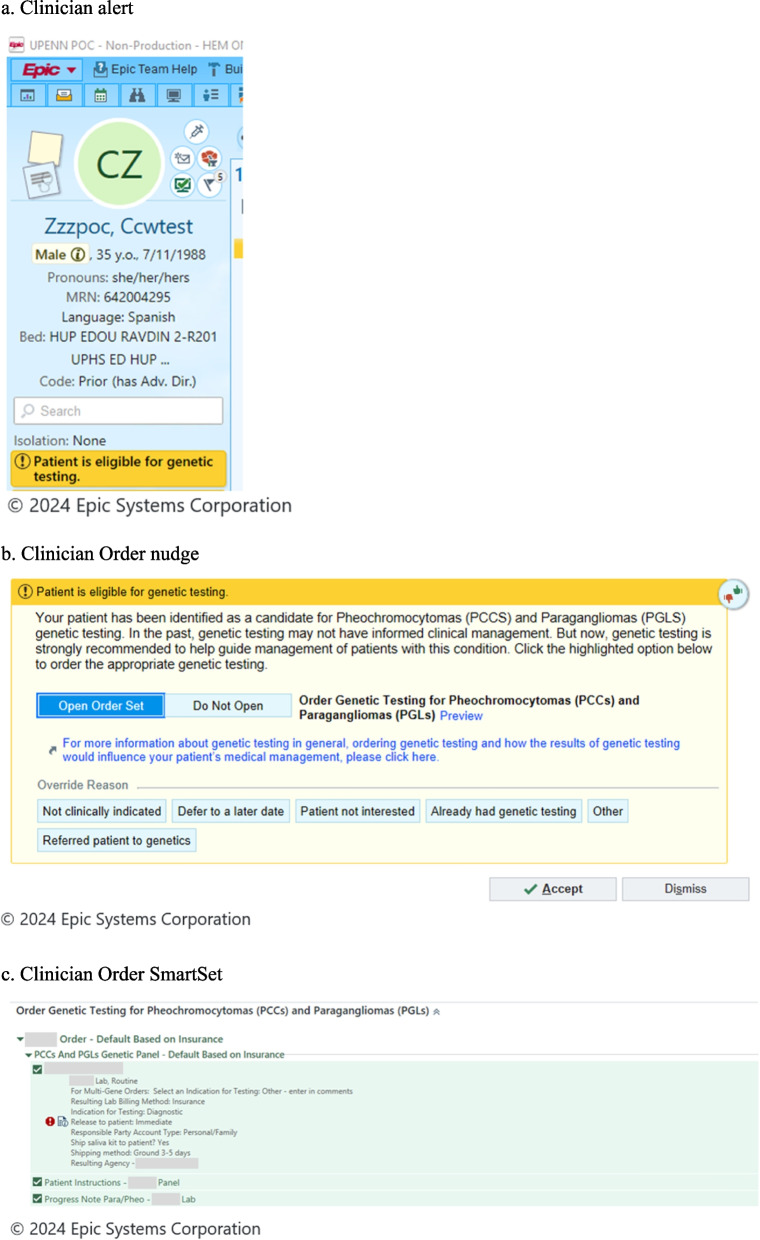

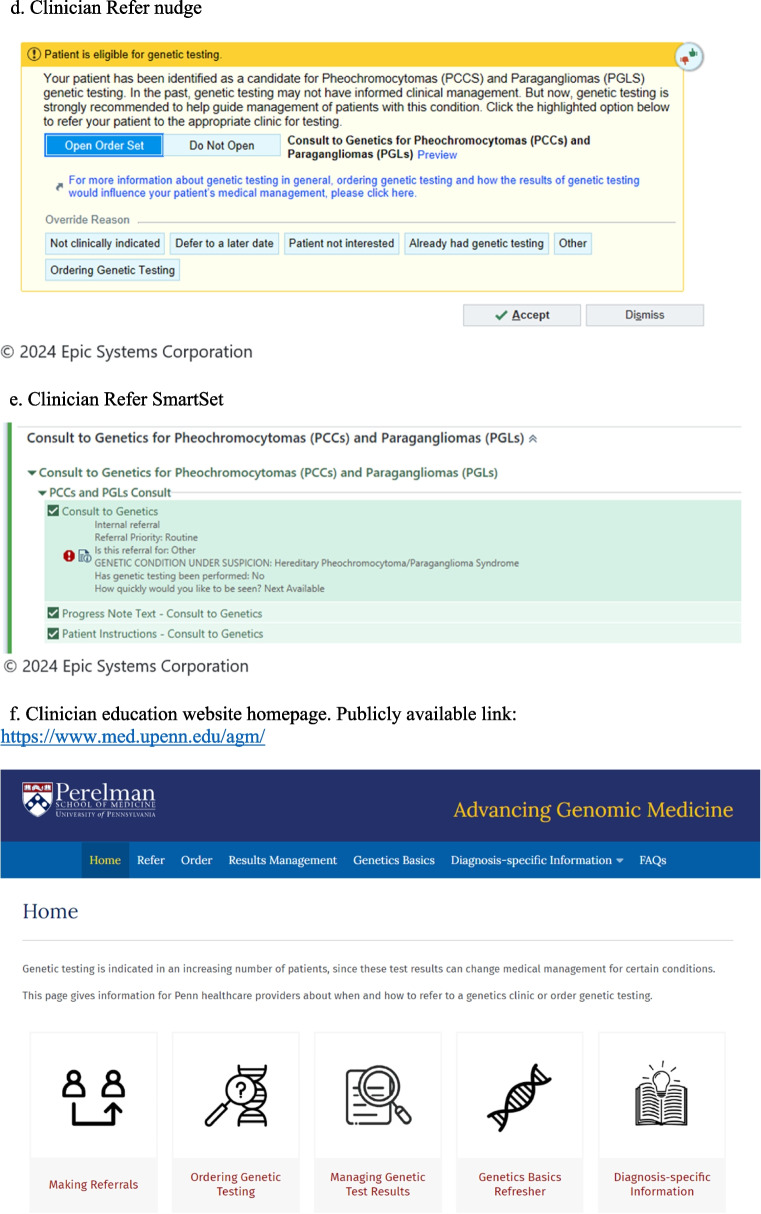
Clinician Nudge. **a** Clinician alert. **b** Clinician Order nudge. **c** Clinician Order SmartSet. **d** Clinician Refer nudge. **e** Clinician Refer SmartSet. **f** Clinician education website homepage. Publicly available link: https://www.med.upenn.edu/agm/

Once identified through electronic phenotyping, patients are randomized to patient nudge or no nudge. Patients who already have had genetic testing for the condition of interest will be excluded. Depending on randomization group, clinicians will receive a nudge in the form of an alert prompting them to “Refer”, or place a referral to the appropriate genetics clinic, or “Order” action, meaning order genetic testing themselves, as viewed in Fig. 3. Randomization of clinicians was done by first grouping clinicians who work together into clusters using the “group_id” network analysis routine in Stata 18. Clusters were then randomly assigned without replacement to one of the two clinician nudge arms or to usual care (no nudge). The alerts will activate at the time of the patient visit when the clinician opens the patient’s EHR. These alerts are static and on the ‘storyboard’ component of the chart, so the provider can view the alert throughout the visit, independent of the tab that is open (Fig. 3). The alerts may transition to interruptive (pop-up) alerts should there be a lack of engagement during the pilot phase of the study.

**Fig. 4:**
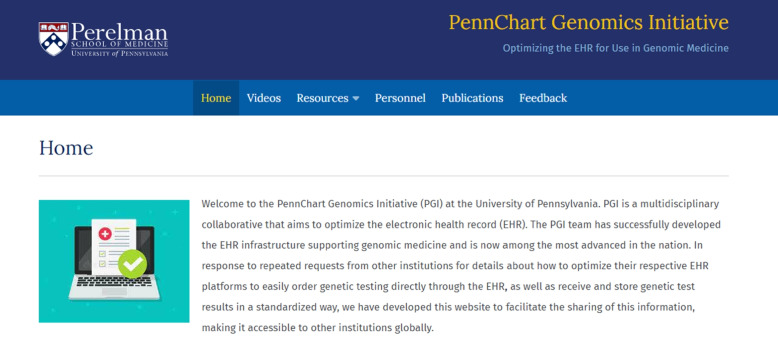
PennChart Genomics Initiative website. Publicly available link: https://www.med.upenn.edu/pgi/

Patients, depending on randomization arm, will receive a nudge to discuss genetic testing with their clinician through the Penn Way to Health program, an evidence-based patient engagement platform used in ongoing studies [87, 88]. Patients will receive a text message around one week prior to their appointment, as well as around three days prior to the appointment (Fig. 4).

Moderators of effectiveness will be investigated through analyzing associations with variables available via EHR for patients (e.g., race) and clinicians (e.g., location of practice). Tracking referrals and genetic test orders in the EHR will allow for comparisons in uptake across clinics and patient groups.

The primary implementation outcome will be the time to genetic testing within six months of the patient’s initial visit with a randomized clinician. Each of the six randomization groups, as detailed in Fig. 2, will be analyzed separately.

### Study setting, population, and duration

The study setting for this project will be specialty clinics at sites within Penn Medicine including: the Hospital of the University of Pennsylvania (HUP), Penn Presbyterian Medical Center (PPMC), Pennsylvania Hospital, Chester County Hospital, Penn Medicine Princeton Health, and Lancaster General Hospital (LGH). The study was approved by the University of Pennsylvania Institutional Review Board. The trial presents minimal risks to participants, and a waiver of informed consent was approved for all study aims.

#### Clinician selection

Around 230 specialty clinicians (physicians and APPs) were identified who may see patients with the conditions selected for the trial, and therefore may be eligible to receive a nudge that their patient is eligible for genetic testing. Clinicians selected must 1) be currently in practice at Penn Medicine; and 2) have prescribing authority in Pennsylvania (physician or APP). Clinician clusters were formed to reduce confounding and randomized within clinical subspecialty (i.e. clinicians seeing patients within each of three disease groupings to be included in the trial: 1) pheochromocytoma/paraganglioma, 2) cardiology conditions, and 3) neurology conditions).

#### Patient selection

Patients will be selected by using the following parameters: 1) patient has a condition included in the trial and is identified through an electronic phenotyping algorithm (Supplemental Table 1); 2) patient is over the age of 18 years old; 3) patient has been seen for two encounters with the condition within the last three years; and 4) patient is seeing a clinician identified for randomization. The details of electronic phenotyping algorithms used for this project, which include ICD-10 codes, laboratory measurements, and medication data, will be published separately. Electronic phenotyping methods were validated using manual chart review of sets of randomly selected patient charts to ensure correct identification of patients eligible for the trial. A positive predictive value of at least 85% is required before finalizing any phenotyping algorithm. The EHR algorithms also exclude patients who have previously received genetic testing.

#### Duration

We expect the trial will take three years to complete and will include at least 3,800 patients across the 10 conditions selected. Clinicians and patients will be observed for one year after they are randomized to assess the ordering, referring, and use of genetic testing, and changes to medical management based on genetic test results.

### Overview of rapid cycle approaches and nudge development

Prior to initiating the implementation strategies of this trial, rapid cycle approaches (RCAs) were used to learn and adapt from pilot data. These approaches have been used in prior work of the Penn Implementation Science Center in Cancer Control (ISC3) [87, 88] and help to de-risk and optimize nudges as implementation strategies. The iterative processes used are summarized in Table 1.

**Table 1:** Rapid cycle approaches to develop implementation strategies

**Domain**	**Approach**	**Iterative Work**	**Output**
Clinician Nudges	*Refer Nudge*: Alert with SmartSet containing: • Order for consult to the corresponding genetics clinic • Language to add to progress note and patient after visit summary regarding genetic testing *Order Nudge*: Alert with SmartSet containing: • Order for genetic testing with preselected gene panel(s), and insurance-sensitive • Language to add to progress note and patient after visit summary regarding genetic testing	Key Questions: • What are the key cognitive heuristics affecting genetic testing uptake? • What support is most helpful to provide clinicians referring to genetics or ordering genetic testing? Method: Discussions with clinicians, discrete choice experiment, legal review of after visit summary language regarding genetic testing. Key feedback: • Status quo bias was a key barrier. • Depending on the state, signed consent for genetic testing is not required; documentation of discussion of genetic testing in the progress note is sufficient (e.g. in Pennsylvania). • Clinicians request support most in return of results.	• Alerts with link to informational website for clinicians. • Clinician website link provided at the time of genetics consult or genetic test order, and upon results return in the EHR.
Patient Nudges	Nudge delivered to patients	Key questions: • What are the key cognitive heuristics affecting genetic testing uptake? • What support is most helpful to provide patients to discuss genetic testing with their clinicians? • What strategies can be used to overcome inequities in patient portal access? Method: Clinician review, patient focus groups (two groups of five patients each). Key feedback: • The message targeting focusing effect bias was preferred within focus groups. • Creation of an educational video about genetic testing was recommended by patients. • Text messaging through Way to Health platform determined to be the best option to avoid disparities in patient portal access.	• Patient text messages developed and will be sent two times prior to appointment with specialty clinician (around seven days and three days prior). • Patient-facing animated explainer video (around two minutes long) created to be sent with patient nudges. • Patient-facing text messages and video will be translated into Spanish.

For development of the clinician nudges, we conducted a discrete choice experiment (DCE) with clinicians who provide genetic testing across the health system to test four messages, each focused on addressing a different potential heuristic, informed by behavioral economics (status quo, focusing effect, impact, and omission), that may be associated with willingness to refer to genetics or order genetic testing. Clinicians were asked their preference between each pairing of messages and in both orders. Of the 79 clinicians approached, 43 (54%) completed the DCE. Preference was significantly greater for the message that addresses status quo bias (44%), compared to focusing effect (22%), impact (18%), or omission (17%) bias (χ^2^ [3]=46.17, *p*<0.001). Preference did not vary by clinician sex or academic rank (*p*’s >0.05).

Template patient nudges were pilot tested by presenting them to two focus groups comprised of five patients and family members per group. Patients were identified by clinicians involved in the trial as having a diagnosis being considered for the trial. A principal investigator moderated videoconference discussion of patient attitudes towards the four nudges. The discussions were transcribed and evaluated for themes. Based on extensive review from the focus groups, study partners, and external advisory partners, the nudges to be implemented in the study were designed as shown in Figs. 3 and 4.

### Patient nudges and support

The patient nudge is designed to “prime” the patient to discuss the potential benefits of genetic testing with their clinician at the time of their next appointment. The patient nudge will be delivered via text message directly to the patient’s cell phone through the Penn Way to Health program. Text messaging was selected as the mode of nudge delivery since an estimated 25% of patients do not use the patient portal as a conduit for health information. National survey data identified that compared to White individuals, Black and Hispanic individuals were less likely to be offered patient portal access and among those offered, were also less likely to access their portal [89]. If sent, the patient nudge will be delivered around one week prior to their medical appointment, and then again around three days prior to their appointment. Additionally, the patient nudge will contain a link to a two-minute informational video providing basic background on genetic testing created as part of this trial (Fig. 4).

### Clinician nudges and support

#### Clinician nudge to refer

For the “Refer” clinician nudge, if accepted, a customized workflow in the form of an order set, called an Epic SmartSet, will appear for the clinician which includes a consult order for the appropriate genetics clinic (determined by electronic phenotyping), and text to add to the clinic note and the patient’s after visit summary about the plan for referral to genetics. Once the consult to a genetics clinic is placed, that clinic will reach out to the patient directly to schedule a visit.

#### Clinician nudge to order

For the “Order” clinician nudge, the Epic SmartSet function also will be utilized. The SmartSet will include 1) genetic testing order with a default set of condition-specific genes and a testing lab selected; 2) Epic Smartphrase to populate the clinician’s note regarding the discussion and plan for genetic testing; and 3) Epic Smartphrase to populate the patient’s after visit summary with information about genetic testing. The list of genes included in the testing is based on guidelines and condition expert recommendation. The selection of the testing laboratory also will be sensitive to the patient’s insurance, so if the patient is capitated to a certain commercial testing laboratory that offers appropriate testing, the testing will go to that laboratory. Genetic testing information text for patients was developed with input from study partners and approved by the legal team (Supplemental Figure 2). The one-page text contains language about the possibility of different types of genetic test results (positive, negative, variant of uncertain significance), implications for family members, guidance on how to learn more about test cost and lab billing policies, and other insurance implications including the Genetic Information Non-discrimination Act (GINA) [90, 91]

The clinician nudges also contain a link for an informational website, designed to provide clinician-facing information about genetic testing, processes to place referrals to genetics, order genetic testing, results management, and frequently asked questions. Screenshots of the nudges, Epic SmartSets, and the homepage of the provider informational website are included in Fig. 3.

#### Nudge to patient and clinician (refer or order)

The arms with patient and clinician nudges encompassing the steps outlined above.

#### Procedures and measures

To launch the nudges, a multi-phase approach will be used. EHR phenotyping algorithms will first be tested by conducting chart reviews. Initially, 20 charts will be reviewed to confirm a patient identified by the algorithm has the targeted diagnosis. Next, 100 charts will be reviewed, and the algorithm will be refined until achieving at least 85% accuracy. This process is iterative until an 85% positive predictive value is reached. In the next phase, a clinician alert will fire invisibly in the background of the EHR without prompting nudges to patients or clinicians. The results of these background alerts will be evaluated to verify accuracy and adjust the EHR algorithm as needed. Subsequently, the clinician nudges will be activated for several weeks among a limited number of clinicians. We will compare the patients identified through the algorithm and confirm that those patients and clinicians who should have received nudges are receiving them. We will also solicit feedback from clinicians on the acceptability of the alerts. Nudge delivery will be monitored in all arms throughout the trial.

The primary measure of implementation is time to genetic testing. The clock begins running with the first (index) visit to a randomized clinician, and ends with either a testing event, or censorship at six months. Those patients who may have multiple visits and cross from a clinician randomized in one group (order nudge, refer nudge or no nudge) to a clinician randomized to a different group will be censored out of the primary analysis at the time of crossover. However, observation will continue out to the end of six months, and a secondary analysis may include those exposures to different clinician arms. Secondary measures of implementation will be the time to genetics referrals and genetic test orders, defined as referrals to genetics placed, and orders for genetic testing, completed or not. Patient engagement rate will be assessed by the frequency of patient responses to text messages, as well as views of the genetic testing informational video. Patient information (e.g., sex, race, condition) will be ascertained from the EHR.

#### Sample size, power, and statistical analysis

Based on preliminary data, up to 3800 patients are anticipated to be available to include in the study (based on prior assessment of eligible patients over the past three years), interacting with approximately 230 clinicians represented by approximately 100 clinician clusters. Clinician nudge assignment will be randomized by clinician cluster, while patient nudges will be assigned independently. This independent randomization makes the main effect for patient nudges a within-cluster comparison. The analysis will use the time to genetic testing outcome registered in the EHR within six months after the visit when the nudges were delivered. The exchangeable correlation (for our clustered data) observed from other studies is small (0.07). Our goal is to achieve at least 80% power using a two-sided type-1 error of 5%. Based on prior data, our outcome is expected to be 10% in the absence of intervention by the 6-month censoring time. We anticipate that the patient nudge will increase testing by 5% (HR=1.54), and clinician nudges will increase testing by 10% (HR=2.12). In addition, the two nudges together may increase testing more than the strict additive main effects and add up to an additional 10% testing (25% total increase). We calculated power by simulating random data sets in Stata 18. We assumed an exponential daily hazard with event times correlated within cluster and fitted those data sets with aCox regression model. Model variances were corrected using cluster-correlation. Our design yields more than 95% power for the patient and provider nudges regardless of correlation. It also provides at least 90% power to detect (within-between) interactions. Power remains above 80% for a wide variety of departures from our original assumptions. Power for the main effect of provider nudges may drop below 80% for high within-subject correlations (above 0.2), and overall power to detect all differences is reduced as the baseline hazard increases (beyond 20% testing by 6 months).

Time to event outcomes (incidence of testing, incidence of ordering, incidence of referring, incidence of clinician action) will be analyzed using Cox regression, censoring at the six-month end of observation. The study design is factorial, and models will contain categorical predictor terms for clinician (refer vs. order vs. control) and/or patient nudge vs. control. We will include adjustments for fixed effects of site. We will control for type 1 error inflation by hierarchical testing, starting with the overall model significance, followed by overall effects of clinician nudges and then patient nudges. Once we have fitted the main effects model, we will test for interaction between clinician and patient nudges and retain that interaction term if significant (alpha=5%). We will test the two clinician nudges separately and combine them if there are no significant differences, and use a similar approach for other study outcomes using GLM (or GEE for repeated measures) using the appropriate distribution family.

While our design is factorial, with the presumption that a patient will remain in one clinician cluster, we recognize that there will be some movement or crossover between clinician clusters and treatments. For that reason, our primary analysis will censor patient observations at the point of crossover. We will also conduct a secondary analysis “as treated”, using time-varying covariates to represent the accumulated clinician nudge exposures over time.

Next, we will examine variability in our outcomes by treatment arm and moderators (patient-, clinician-, and system-level) using interaction terms within the regression. We will fit an adjusted model using the same approach described in the primary analysis. Moderators of particular interest will be patient-level (e.g., diagnosis, race, geocoded indicator of socio-economic status), clinician-level (e.g., years in practice), and system-level data (e.g., community vs. hospital-based). If there are no interactions with the two clinician nudge arms, they will be combined in subsequent moderator analysis following the same approach described above.

## Discussion

This study is one of the first randomized controlled trials studying the effect of clinician and patient nudges on the uptake of genetic testing for conditions for which this testing is considered standard of care by guidelines and/or expert opinion and results could change medical management. Using the EHR to extract information to inform electronic phenotyping algorithms, patients with conditions of interest will be identified. Nudges linked to EHR-based clinician supports, including Epic SmartSets and links to a clinician educational website, will be used to encourage use of genomic medicine. This work adds to previous studies of nudge implementation to patients and clinicians and applies these strategies to populations in which we have demonstrated underutilization of and disparities in genetic testing. Increasing the uptake of genetic testing is likely to have downstream effects on the uptake of appropriate surveillance and treatment options for patients.

We have included diverse clinics, across specialties, and health care settings, within the University of Pennsylvania Health System with a single EHR (Epic; PennChart and LGHealth) with active efforts to innovate and improve genomics integration. The results of this study may not be generalizable to sites without similar EHR capabilities and clinical genetics programmatic expertise. If these strategies prove beneficial in improving genetic testing uptake and ultimately clinical care for patients, these methods could serve as a model for sustained change in clinical care practices, for other diseases and other health systems. As the study materials are developed and finalized, we will engage in dissemination activities to increase capacity of other medical sites to adopt EHR-based strategies and other implementation strategies used in this trial.

### Supplementary Information

Additional file 1: Supplemental Table 1. Conditions selected for trial and rationale.

Additional file 2: Supplemental Figure 1. Patient genetic test information sheet. 

## Data Availability

Not applicable.

## Abbreviations

APP: Advanced Practice Provider
CDS: Clinical Decision Support
DCE: Discrete Choice Experiment
EHR: Electronic Health Record
FDA: Food and Drug Administration
GINA: Genetic Information Non-Discrimination Act
HL7: Health Level 7
ISC3: Implementation Science Center in Cancer Control
NCCN: National Comprehensive Cancer Network
PPV: Positive Predictive Value
RCA: Rapid Cycle Approaches
RCT: Randomized Controlled Trial
