# Supplementary material for: Protocol for a type 3 hybrid implementation cluster randomized clinical trial to evaluate the effect of patient and clinician nudges to advance the use of genomic medicine across a diverse health system
Source: Implement Sci. 2024 Aug 19;19:61. doi: 10.1186/s13012-024-01385-5 (PMC11331805; doi:10.1186/s13012-024-01385-5)
Supplement: Supplementary file 1 — Additional file 1: Supplemental Table 1. Conditions selected for trial and rationale. [file 13012_2024_1385_MOESM1_ESM.docx]

**Supplemental Table 1**. Conditions selected for trial and rationale.

| **Clinical Domain** | **Condition** | **Selected indications for genetic testing^1^** | **Rationale^2^** | **Supporting Evidence** |
| --- | --- | --- | --- | --- |
| Cardiology | Long QT Syndrome | - All patients | - Possible treatment implications e.g. Beta blockers - Management changes e.g. exercise restrictions, implantable defibrillator placement, ablation | - Expert opinion, guidelines (3, 7) |
|  | Arrhythmogenic cardiomyopathy | - All patients | - Possible treatment implications e.g. myosin modulators, enzyme replacement therapy - Management guidelines - Potential clinical trial eligibility | - Expert opinion, guidelines (3, 7) |
|  | Non-ischemic cardiomyopathy | - All patients | - Possible treatment implications e.g. myosin modulators, enzyme replacement therapy - Management guidelines - Potential clinical trial eligibility | - Expert opinion, guidelines (3, 7) |
|  | Thoracic aortic aneurysm and dissection | - Ascending aortic diameter > 5cm | - Possible treatment implications e.g. Beta blockers, angiotensin receptor blockade - Differing surgical thresholds | - Expert opinion, guidelines (3, 7) |
| Neurology | Alzheimer’s Disease | - Onset < 60 years^1^ | - Diagnosis confirmation - Potential clinical trial enrollment, development of precision therapies | - Guidelines (2) |
|  | Amyotrophic lateral sclerosis | - All patients | - Targeted treatment available (e.g. FDA-approved tofersen, anti-sense oligonucleotide for *SOD1*-related ALS) - Potential Clinical trial enrollment | - Guidelines (2) |
|  | Frontotemporal dementia | - All patients | - Diagnosis confirmation - Potential clinical trial enrollment, development of precision therapies | - Guidelines (2) |
|  | Myopathy | - All patients | - Several approved precision treatments (e.g. exon-skipping antisense oligonucleotide therapy for Duchenne Muscular Dystrophy, enzyme replacement therapy for Pompe disease) - Potential clinical trial enrollment | - Guidelines (2) |
|  | Parkinson’s Disease**^1^** | - Onset <50 years^1^ | - Clinical trial enrollment (e.g. *LRRK2* inhibition and ASO therapy trials) | - Guidelines (2) |
| Tumor predisposition | Pheochromocytomas and paragangliomas | - All patients | - Increased surveillance for additional tumor development - Annual catecholamines and metanephrines | - Guidelines (10) |

^1^There are additional indications for genetic testing (e.g. the presence of family history, or additional diagnoses). However, at this time, these characteristics are either not currently entered in the EHR reliably or are not specific enough to improve the positive predictive value of EHR phenotyping.

^2^Identification of a hereditary predisposition would enable more detailed family counseling and potential cascade screening for each of these conditions
