# Supplementary figures and images for: Protocol for a type 3 hybrid implementation cluster randomized clinical trial to evaluate the effect of patient and clinician nudges to advance the use of genomic medicine across a diverse health system
Source: Implement Sci. 2024 Aug 19;19:61. doi: 10.1186/s13012-024-01385-5 (PMC11331805; doi:10.1186/s13012-024-01385-5)

**Supplemental Figure 1. Patient genetic test information sheet.**

**
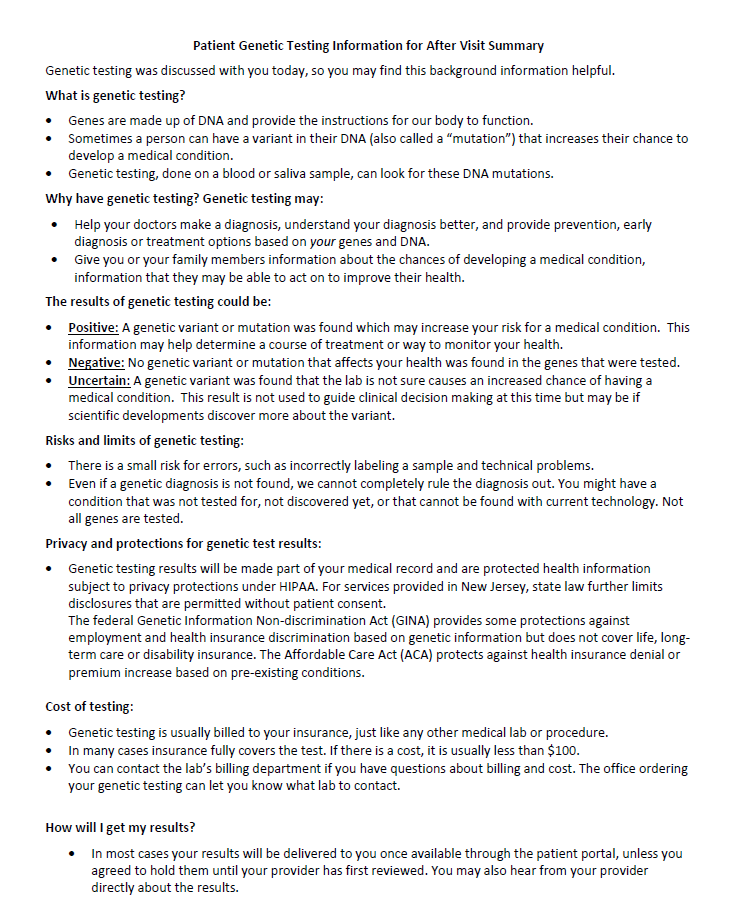
**

Supplement: Supplementary file 2 — Additional file 2: Supplemental Figure 1. Patient genetic test information sheet. [file 13012_2024_1385_MOESM2_ESM.docx]
